# Mode-locking behavior of Izhikevich neurons under periodic external forcing

**DOI:** 10.1186/1471-2202-16-S1-P140

**Published:** 2015-12-18

**Authors:** AmirAli Farokhniaee, Edward W Large

**Affiliations:** 1 Department of Physics, University of Connecticut, Storrs, CT, 06268, USA; 2 Department of Psychology, University of Connecticut, Storrs, CT, 06268, USA

## 

Many neurons in the auditory system of the brain must encode amplitude variations of a periodic signal. These neurons under periodic stimulation display rich dynamical states including mode-locking and chaotic responses [1]. Periodic stimuli such as sinusoidal waves and amplitude modulated (AM) sounds can lead to various forms of *n:m *mode-locked states, similar to the mode-locking phenomenon in a LASER resonance cavity. Obtaining Arnold tongues provides useful insight into the organization of mode-locking behavior of neurons under periodic forcing. In this study we obtained the regions of existence of various mode-locked states on the frequency-amplitude plane, which are called Arnold tongues, for Izhikevich neurons (see Figure 1). This study is based on the model for neurons by Izhikevich (2003), which is a reduced model of a Hodgkin-Huxley neuron [2]. This model is much simpler in terms of the dimension of the coupled non-linear differential equations compared to other existing models, but excellent for generating the complex spiking patterns observed in real neurons [3]. Hence we can describe the construction of harmonic and sub-harmonic responses in the early processing stages of the auditory system, such as the auditory nerve and cochlear nucleus.

**Figure 1:**
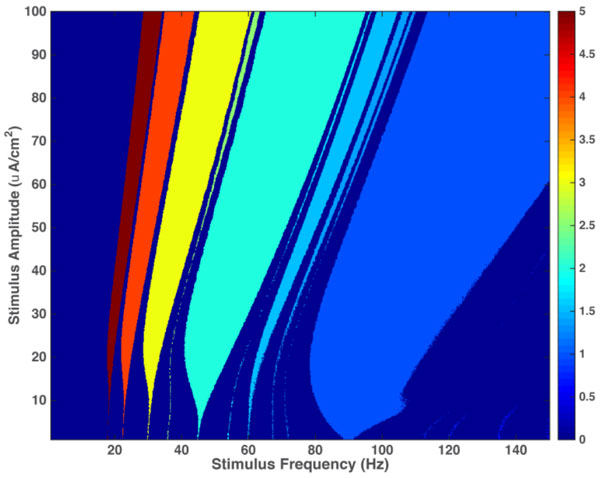
**The Arnold tongues diagram for a Class 1 Izhikevich neuron driven by an external sinusoidal forcing**. This plot represents the mode-locked regions as a function of the amplitude and frequency of this sinusoidal forcing. Each colored region represents a different phase-locked regime in terms of an integer ratio. Here, an *n:m *ratio means the neuron produces *n *action potentials in response to every *m *cycles of the stimulus. For example, for stimulus amplitudes and frequencies corresponding to the yellow region, the neuron exhibits 3:1 mode-locking.

## Conclusion

Different mode-locked regions that are shown in the Arnold tongues diagram are predictors of mode-locking of auditory system neurons to sound, which in turn predict the formation of harmonics and sub-harmonics of the sound in the brain.
